# Merkel Cell Carcinoma of the Retroperitoneum with No Identifiable Primary Site

**DOI:** 10.1155/2013/131695

**Published:** 2013-09-01

**Authors:** Daniele Rossini, Salvatore Caponnetto, Vittoria Lapadula, Lucilla De Filippis, Gabriella Del Bene, Alessandra Emiliani, Flavia Longo

**Affiliations:** Department of Clinical Oncology A, Sapienza University of Rome, Policlinico Umberto Primo, Viale Regina Elena 324, 00161 Rome, Italy

## Abstract

Merkel cell carcinoma (MCC) is an extremely rare primary neuroendocrine neoplasm of the skin that shows aggressive behavior and a poor prognosis. We report a case of a 67-year-old male with a Merkel cell carcinoma which initially presented itself as a large retroperitoneal mass. Pathological and immunohistochemical analysis revealed tissue consistent with neuroendocrine carcinoma. Despite complete medical workup, no other primary MCC could be detected. While being an atypical presentation, the tumor mass showed an excellent response to the combination of chemotherapy followed by radiotherapy.

## 1. Introduction

Merkel cell carcinoma (MCC) is an aggressive neuroendocrine malignancy arising from the skin, with estimated incidence of 0.6 per 100,000 person/years [1]. The incidence appears to be on the rise, due likely to improved diagnostic techniques, increased exposure to known risk factors, immunosuppression, prolonged sun exposure, and a history of prior malignancy [2–4].

The natural history of MCC is unpredictable, ranging from a localized indolent course to a regionally more aggressive course with widespread metastases. Several large reviews document the development of local recurrence in 25–30% of all cases of MCC, regional disease in 52–59% of all cases, and distant metastatic disease in 34–36% of all cases [5, 6].

On examination, the clinician should focus on the lymphatic and integumentary systems [7]. When symptoms lead to suspicion of recurrence, appropriate imaging studies should be performed. The aggressive nature of this disease necessitates frequent followup, and the presence of risk factors including tumors larger than 2 cm, truncal location, male sex, age over 65, nodal or distant disease at presentation, and duration of disease before presentation [8] should determine the appropriate frequency. Also, in this type of tumor, the stage of disease stratifies the rate of survival groups. For example, according to Albores-Saavedra et al., the 10-year relative survival rate of localized stage MCC is 71%, whereas the 10-year relative survival rates for regional and distant stages are 47.8% and 20.1%, respectively [1].

Most Merkel cell carcinomas develop within the skin of the head and neck or on the extremities. In a review of 661 cases conducted in 2000, only 2 percent presented with no apparent primary lesion [8].

## 2. Case Presentation

**Figure 1:**
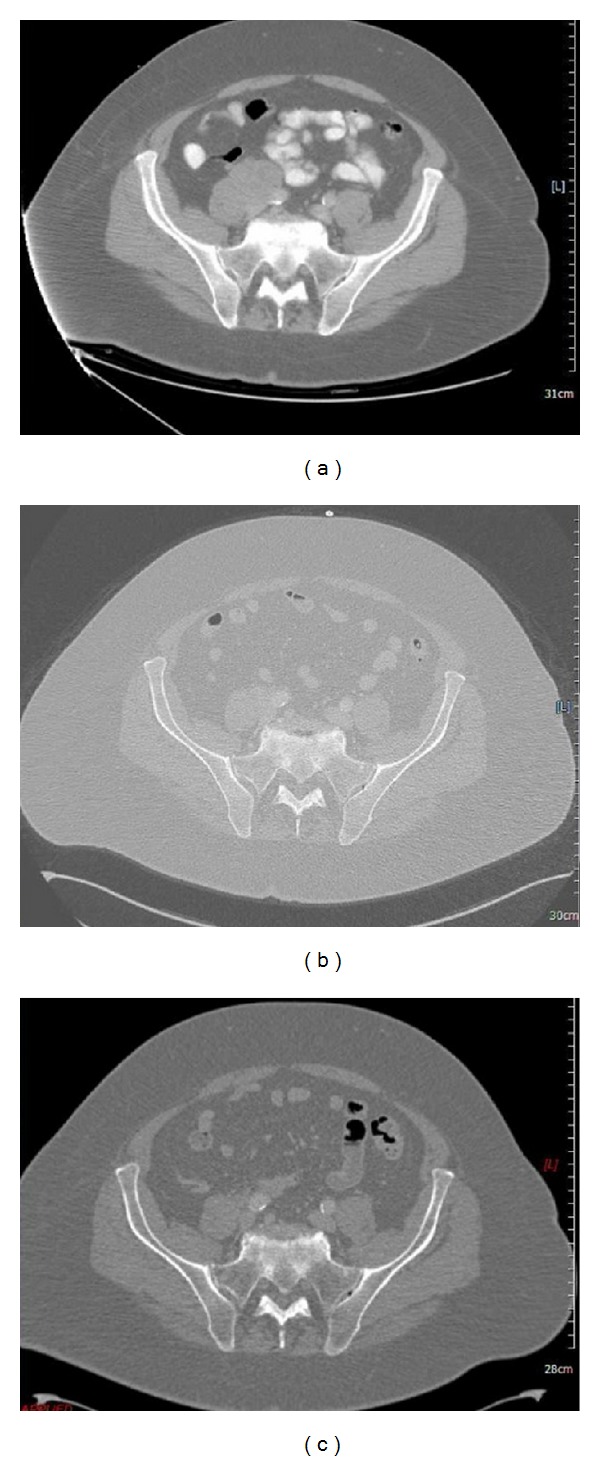
CT scan of pelvis showing right mass at diagnosis, after-chemotherapy and after-radiotherapy.

A 67-year-old man, with a history of hypertension, obesity, hypercholesterolemia, and paroxysmal tachycardia, presented with a firm and palpable mass in the right inguinal area without any skin lesions. The ultrasound findings showed a massive and well-circumscribed nodular hypoechoic lymph node formation with a diameter of 4.7 × 3.5 cm. A second similar formation, large 3.8 × 3.1 cm, was at the root of the thigh. An excision biopsy was performed: focal areas due to residual nodal structure, site of metastases from malignant neoplasm composed of nests, cords, and sheets of elements of medium size with rounded nuclei, finely dispersed chromatin and scant cytoplasm. In the tissue there is also the presence of large necrotic areas with images of vascular invasion. Phenotype of neoplastic cells was as follows: panCK (MNF116) + (do-like), CK20 + (dot-like), neurofilament + (dot-like), synaptophysin +/−, NCAM +/−, chromogranin−, CK7−, CD99−, TTF1−, and CD45−. These findings were consistent with the diagnosis of a metastatic MCC. A subsequent CT Whole Body scan (Figure 1) showed at right common iliac level a new formation of pathological tissue with irregular margins, with markedly heterogeneous contrast enhancement and areas of central necrosis of 45 mm which has a longitudinal extent of about 67 mm. This lesion appears inseparable from the psoas muscle and the ipsilateral iliac vessels. At the height of the thigh, a package of 83 × 60 mm was observed which was associated with marked thickening of adipose tissue around the wound, edema, and multiple other small lymphadenopathies. Here, there was also a left inguinal lymph node of probable nonspecific appearance of 20 mm. Thickening of adipose tissue combines multiple mesenteric lymph nodes of 15 mm in diameter, while other lymph nodes were in the para-aortic area.

Given the results obtained and the clinical state of the patient, it was decided to start treatment according to the following schema: carboplatin (CBDCA) (300 mg/m (2) of AUC 5 on day 1) and etoposide (VP-16) (100 mg/m (2) on days 1–3) every 3 weeks for a total of six cycles. After 3 doses, a new reevaluation with CT was performed: the pathological lymphadenopathy reported markedly reduced in any size in the right iliac between the psoas muscle and the iliac vessels, in the surgical scar in the groin (30 mm versus 45 mm), and at level of the root of the right thigh (46 × 25 mm, prev. 83 mm × 60 mm). Also, the edematous thickening of adipose tissue appeared modestly decreased, and the small lymphadenopathy context was reduced in number and size.

The posttreatment CT of total body (Figure 1) showed in the abdomen/pelvis a reduction in the size of the pathological lymphadenopathy reported previously in the right iliac between the muscle psoas and iliac vessels (19 versus 21 mm) and at the level of the root of the right thigh (versus 26 × 16. 46 × 25 mm); localized lymphadenopathy in the groin at the surgical scar was unchanged. The edematous thickening of adipose tissue and small contextual lymphadenopathy at this level was further reduced.

Given the results obtained, a surgical evaluation was requested which excluded the surgery for cardiac comorbidities.

Radiotherapy on the right abdominal inguinal region (200 cGy/day for 5 days/week) + boost on the previous area neoplastic to a total dose of 68 Gy was then performed.

A reevaluation with total-body CT (Figure 1) showed a reduction in size of the pathological lymphadenopathy previously reported in the right iliac (2.2 versus 3 cm). The lymph node described at the root of the right thigh was thinner and had a lower contrast enhancement compared to the previous. The edematous thickening of adipose tissue and contextual small lymphadenopathy was also reduced.

## 3. Discussion

 According to the large population study by Albores-Saavedra et al. of the majority, (97.6%) cases of MCC occurred in the skin. Among the less common sites are the external surface of the lip (0.4%), the parotid gland (0.4%), the submandibular gland (0.1%), the nasal cavity (0.1%) and the lymph nodes, vulva, vagina, and esophagus (<0.1%) [1].

As far as we know, in the literature, there are rare and occasional cases of Merkel cell tumors that originate from the retroperitoneum [7, 9], but strong suspicion remains that these are the expressions of a metastasized tumor whose primitive declined.

We have to remember in fact that it is difficult to diagnose MCC in a lymph node due to its similarity to other poorly differentiated small basophilic cell tumors, including metastatic melanoma, lymphoma, small cell carcinoma, and neuroendocrine carcinoma metastatic from other organs [10]. Immunohistochemistry is necessary for the definitive diagnosis. In this case, the tumor cells were positive for cytokeratin 20 and CD56, which demonstrated both epithelial and neuroendocrine features. The histologic diagnosis was also aided by the finding of a dot-like pattern with CK 20 and CK 7 stainings.

The presentation of this abnormal neoplasia can be explained according to two main justifications, in agreement with what has already been proposed by Boghossian et al. [7]: the mass could be the expression of a replacement of a lymph node with a primitive neoplasia, or an initial skin lesion could have spontaneously regressed leaving the retroperitoneal mass as single site of metastasis. A third hypothesis, although anecdotal, is explained in some studies that suggest a possible onset of Merkel cell tumor in the subcutaneous tissue [11, 12]. It is however interesting to note that our patient, despite the absence of a history of immunosuppression and a presentation not compatible with sun exposure, makes use of statins. The role of these drugs seems controversial, but there are lines of evidence of a possible influence [13].

According to the NCCN guidelines the use of chemotherapy with or without surgery and/or radiation therapy is indicated for stage IV, distant metastatic disease (M1) [14]. The most common regimen used for regional disease is cisplatin or carboplatin with or without etoposide [15]. Although combined treatment with radiotherapy and surgery has a better impact with significantly lower rates of local and regional recurrence of MCC than surgery alone [16], in the presented case, it was not possible to associate the treatments as required due to the history of paroxysmal tachycardia. Combination of carboplatin and etoposide followed by radiotherapy has shown, however, effective in inducing a reduction of the tumor mass with an acceptable toxicological profile.

In the situation described, it clearly emerges, albeit in a unusual presentation, that the treatment of Merkel cell cancer may benefit from systemic chemotherapy based on cisplatin or carboplatin plus etoposide combined with radiation therapy.

Clearly, as there is yet no certainty about the best approach in Merkel cell tumors, further studies are needed to investigate the issue of unusual presentations of this cancer.
